# Study on the effectiveness of implementing case-based learning within the CDIO framework in an “evidence-based nursing” curriculum: a longitudinal study

**DOI:** 10.3389/fmed.2025.1702678

**Published:** 2025-12-03

**Authors:** Wei Luo, Guangyu Yang, Hua Yuan, Yao Lu, Xueqi Dong, Meixue Hou, Hui Xue, Xiuying Zhang

**Affiliations:** 1 Department of Fundamental Nursing, School of Nursing, Jilin University, Changchun, China; 2 Department of Otorhinolaryngology Head and Neck Surgery, The First Hospital of Jilin University, Changchun, China; 3 Department of Histology & Embryology, College of Basic Medical Sciences, Jilin University, Changchun, China

**Keywords:** evidence-based nursing, case-based learning, emotions, control-value theory, learning process

## Abstract

**Background:**

Evidence-based nursing curriculum is a key course to develop nursing students’ evidence-based practice competencies. How to plan and design an evidence-based nursing curriculum to improve students’ competencies has been an issue explored by nursing educators. Case-based learning (CBL) has been widely used in teaching practices that promote the development of nursing students’ practice competencies. However, an effective framework for implementing CBL in evidence-based nursing curricula to facilitate the development of these competencies remains unclear. Additionally, most current evaluations of the effectiveness of CBL implementation focus on endpoint indicators, while neglecting the impact on learners’ learning processes. This focus is not conducive to analyzing the mechanisms behind CBL’s effectiveness in student development and hinders the continuous optimization of instructional design.

**Objective:**

This study explored the effectiveness of an instructional model that integrates CBL with the Conceive-Design-Implement-Operate (CDIO) model and analyzed the students’ learning processes based on the control-value theory (CVT).

**Design:**

A longitudinal pre- and post-test study.

**Methods:**

The study was conducted in a master’s nursing program at a university in China, involving 64 students. Participants completed the questionnaires before and after the implementation of the instructional model. The data were analyzed using SPSS 27 software.

**Results:**

Excellent results were achieved in this instructional model. Students’ knowledge and skills increased significantly. Our results also demonstrated that students’ competencies (attitudes, skills, and knowledge) were influenced by control appraisals, emotions, and learning strategies.

**Conclusion:**

Future research should vigorously develop such models to advance evidence-based nursing. Furthermore, educators should focus on key factors that influence student learning to foster a more conducive teaching environment.

## 1 Introduction

Over the past few decades, evidence-based practice has become an important focus for medical practitioners and researchers worldwide. It has been recognized as the gold standard for providing safe and compassionate healthcare (1). Numerous studies have confirmed that the implementation of evidence-based practice was critical to improving patient safety, satisfaction, and clinical outcomes (2–4). However, a systematic review showed that while most nurses were familiar with the concept of evidence-based practice, they felt that their knowledge and skills in this area were inadequate, making them unable to use the best evidence in their practice (5). Similarly, another study pointed out that if evidence-based practice is not effectively implemented by nurses, it may result in patients not achieving better outcomes (6).

Evidence-based nursing curriculum is the foundation for developing nursing students’ and nurses’ evidence-based practice competencies (7). It is also recommended as a core curriculum for nursing education and has long been integrated into undergraduate nursing education systems throughout Europe (8, 9). Currently, evidence-based practice has been incorporated into China’s undergraduate and graduate education programs. Scholars have used various teaching strategies to promote nursing students’ evidence-based practice competencies (10–12). CBL, as a contextualized teaching method, is an effective approach for applying theoretical knowledge to solve practical problems in the classroom (13). Yao et al. (14–16) have conducted extensive work in applying CBL to evidence-based nursing courses for nursing students. They identified key aspects of applying CBL in evidence-based nursing courses: preparation, student engagement, group discussions, and teacher feedback (14). Based on students’ preferences, Yao et al. (15) also identified key attributes that promote their participation in CBL, such as provider, case modality, and group size, and selected the optimal adjustable combination of each attribute according to instructional conditions, providing important empirical evidence for the effective implementation of CBL. Furthermore, Yao et al. (16) concluded through a systematic review that CBL can influence students’ attitudes, skills, and knowledge. However, how to design CBL to enhance students’ evidence-based practice competencies and the possible role mechanisms of CBL in this process remain unclear.

The CDIO is an international initiative in educational reform aimed at improving engineering education globally, and it consists of four phases: Conceive, Design, Implement, and Operate (17). The framework targets competency development and fosters direct student involvement in task design, active learning, seminar participation, and project-oriented group tasks (18). This active learning improves students’ cognitive abilities, professional skills, and learning interests (19, 20). In fact, using CBL in any form in the classroom requires consideration of students’ learning burden, emotions, engagement and learning outcomes to promote the effective application of CBL (15, 21). However, current evaluations of the effectiveness of CBL implementation in evidence-based practice have largely focused on endpoint indicators such as student knowledge, attitudes, and perceptions, without considering the impact on the learning process of learners.

**Figure 1:**
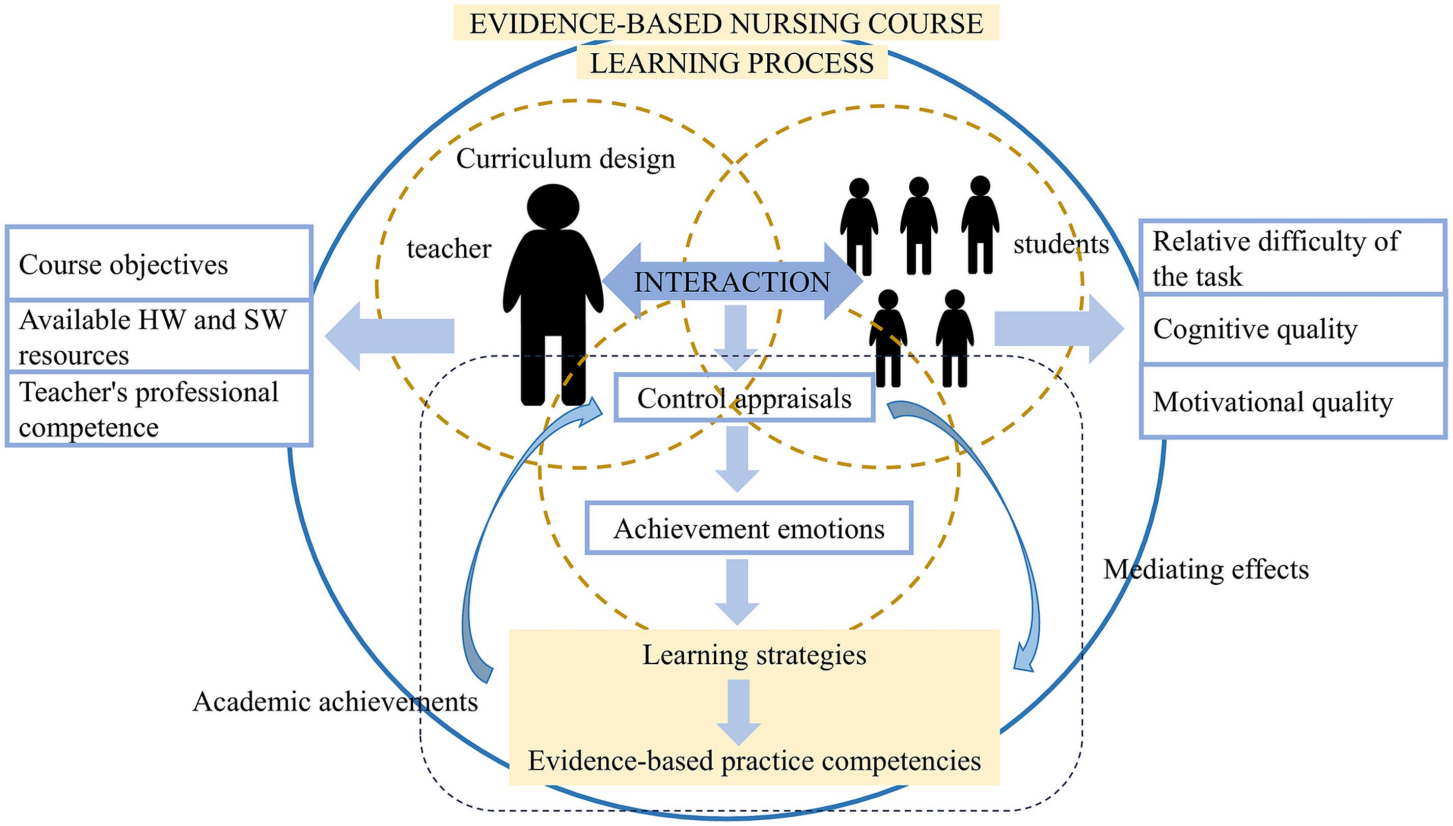
Schematic diagram of hypotheses based on the control-value theory. HW, hardware; SW, software.

The CVT recognizes that the student learning process mainly encompasses four components: environment, appraisal, achievement emotion, and academic achievement (22). According to the CVT, various environmental factors can affect emotions by influencing control appraisals, producing a series of ripple effects, as shown in Figure 1. Control appraisals refer to appraisals of control over actions and outcomes of the learning process (controllability) (23). CVT proposes that specific achievement emotions arise from an individual’s controllability over subjectively important achievement activities or outcomes. Achievement emotions are defined as the affective arousal directly related to achievement activities (23). Positive emotions enhance learning by strengthening motivation, increasing engagement, shaping character, and motivating students to achieve their learning goals, whereas negative emotions can diminish academic performance by weakening these factors (24, 25). CVT also identifies that achievement emotions mediate the relationship between control appraisals and academic achievement, with a reciprocal causality existing between control appraisals and achievement.

Based on the CVT, we explored the formation of learning strategies and evidence-based practice competencies (attitudes, skills, and knowledge). Well-developed learning strategies can promote autonomous and reflective work and facilitate deeper learning, which are necessary skills for evidence-based practice learners undertaking clinical practice. As the future clinical workforce, nursing students, especially graduate nursing students, are expected to develop flexible learning strategies (26) and high-quality evidence-based practice competencies (27) to adequately address the challenges encountered in evidence-based nursing practice. Therefore, this study designed and implemented CBL within the CDIO framework to facilitate the development of students’ learning strategies and evidence-based practice competencies. It also explored the impact of applying this model on learners’ learning processes based on the CVT, aiming to clarify the effectiveness and possible mechanisms of the model in developing students’ evidence-based nursing practice competencies. With this background, we proposed the following hypothesis:

*Hypothesis 1*. In the learning environment of an evidence-based nursing curriculum where the instructional model is applied, students’ achievement emotions can change.

*Hypothesis 2*. In the learning environment of an evidence-based nursing curriculum where the instructional model is applied, control appraisals are associated with achievement emotions, learning strategies, and evidence-based practice competencies.

*Hypothesis 3*. In the learning environment of an evidence-based nursing curriculum where the instructional model is applied, achievement emotions are associated with learning strategies and evidence-based practice competencies.

*Hypothesis 4*. In the learning environment of an evidence-based nursing curriculum where the instructional model is applied, achievement emotions mediate the relationship between control appraisals and both learning strategies and evidence-based practice competencies.

*Hypothesis 5*. In the learning environment of an evidence-based nursing curriculum where the instructional model is applied, evidence-based practice competencies and learning strategies can affect control appraisals.

*Hypothesis 6*. In the learning environment of an evidence-based nursing curriculum where the instructional model is applied, learning strategies can influence evidence-based practice competencies.

## 2 Methods

### 2.1 Study design

A longitudinal pre- and post-test study was conducted to examine the relationship between the variables in this study.

### 2.2 Participant

The participants were first-year students pursuing a master’s degree in nursing at a university in China. The sample size was calculated using G*Power for a paired t-test, with an effect size of 0.5, *α* = 0.05, and power of 0.8, requiring a total of 34 students. Students who met the following criteria were eligible to participate: (1) selected and attended the evidence-based nursing curriculum; (2) possessed reading and comprehension skills. The first survey was conducted by 85 participants, and 70 participants finalized the second survey. We excluded questionnaires that were completed only once, contained errors in basic information, or had excessively short response times, resulting in a final analytical sample of 64 matched responses.

### 2.3 Setting

**Figure 2:**
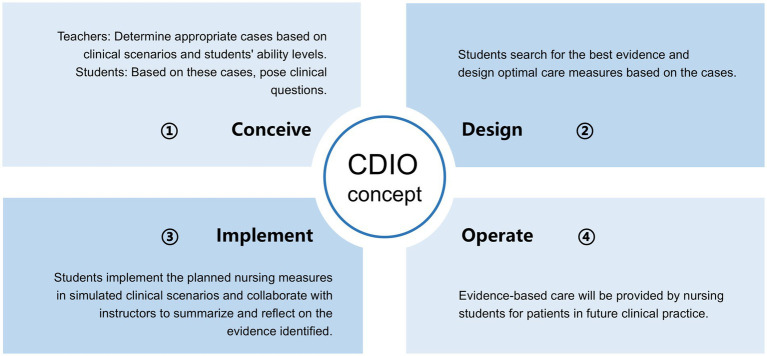
Specific procedures for the application of CDIO framework.

The evidence-based nursing curriculum for this study was designed using a model that integrated CBL and the CDIO framework. The evidence-based nursing course comprises 10 sessions, of which 5 employ this instructional model, each lasting 4 h. It is taught by XYZ and HY, two veteran teachers who have instructed the evidence-based nursing curriculum for 10 to 15 years. Cases were obtained from real clinical scenarios and were selected at the discretion of these two senior faculty members, as well as clinical professionals. This teaching approach comprises the following components: basic knowledge instruction, case selection, group discussion, PPT production, timely feedback, and course reflection. The CDIO framework was implemented in a step-by-step process as the course unfolds. Figure 2 illustrates the specific steps of the CDIO framework application.

### 2.4 Measures

#### 2.4.1 Control appraisals

Control appraisals were measured using 5 items from the Academic Control Scale (28), while the other 3 items were not used. The scale was used in this study for the assessment of academic perceived controllability. Cronbach’s alpha was 0.80. Participants scored on a 5-point Likert scale (1 = strongly disagree, 5 = strongly agree). The example item is “I see myself as largely responsible for my performance throughout my college career.” Cronbach’s alphas for this study are shown in Table 1.

**Table 1:** Descriptive statistics for variables (*N* = 64).

Variable	M1	SD1	M2	SD2	Alpha1	Alpha2	*T*	*P*
CA	3.94	0.53	3.82	0.5	0.74	0.66	1.81	0.08
PA	3.89	0.37	3.93	0.35	0.87	0.86	−1.34	0.18
PD	3.53	0.43	3.58	0.42	0.88	0.87	−1.35	0.18
NA	3.14	0.6	3.12	0.64	0.93	0.94	0.47	0.64
ND	2.61	0.57	2.66	0.58	0.93	0.92	−1.07	0.29
CS	2.79	0.44	2.93	0.59	0.66	0.89	−1.8	0.08
SMS	2.88	0.48	2.93	0.51	0.69	0.75	−0.71	0.48
ELP	2.97	0.41	3.02	0.49	0.63	0.82	−0.85	0.4
EBPA	4.02	0.32	4.07	0.39	0.6	0.72	−1.27	0.21
EBPS	3.28	0.57	3.42	0.57	0.66	0.77	−2.2	<0.05*
EBPK	3.4	0.74	3.8	0.56	0.78	0.63	−4.35	<0.001***
BM	2.57	0.59	2.63	0.6	0.89	0.87	1.28	0.21

CA, control appraisals; PA, positive activating emotions; PD, positive deactivating emotions; NA, negative activating emotions; ND, negative deactivating emotions; CS, comprehension strategies; SMS, self-management strategies; ELP, effort level and persistence; EBPA, evidence-based practice attitudes; EBPS, evidence-based practice skills; EBPK, evidence-based practice knowledge; BM, boredom. *p < 0.05, ***p < 0.001.

#### 2.4.2 Achievement emotions

We measured learners’ achievement emotions in the evidence-based nursing course using 88 items from the General Academic Emotion Questionnaire for College Students (29). Cronbach’s alphas ranged from 0.64 to 0.89. The scale contains 4 dimensions (10 achievement emotions): positive activating emotions (e.g., enjoyment, hope, and interest), positive deactivating emotions (e.g., pride, relaxation), negative activating emotions (e.g., anger, anxiety, and shame), and negative deactivating emotions (e.g., hopelessness, boredom). A Likert scale was used for scoring (1 = strongly disagree, 5 = strongly agree). The example item is “I’m satisfied with my academic performance.”

#### 2.4.3 Learning strategies

To measure students’ learning strategies, we used the PISA Learning Strategies Scale (30). It consists of 12 items across 3 dimensions: comprehension strategies, self-management strategies, and effort level and persistence. The scale demonstrated good reliability and validity. Participants rated their learning strategies on a 4-point Likert scale (1 = almost never, 4 = almost always). An example item is “I can continue to study in the learning process even in the face of difficulties.”

#### 2.4.4 Evidence-based practice competencies

We used 12 attitudes (31), 6 skills, and 3 knowledge from the Evidence-Based Practice Evaluation Competence Questionnaire (32) to measure evidence-based practice competencies. The items for the remaining 3 knowledge were not utilized. Participants responded on a 5-point Likert scale ranging from 1 = does not apply to 5 = fully applies. Cronbach’s alphas for each subscale (attitudes, skills, and knowledge) were 0.94, 0.76, and 0.80. A sample item is “The evidence-based practice helps to make decisions in clinical practice.”

### 2.5 Data collection and analysis

Questionnaires were distributed both before and after the implementation of this teaching model. The first questionnaires were collected from October 30 to November 3, 2023, and the second from November 24 to 27, 2023, until there were no more participants. The SPSS 27 software program was used to analyze the data. Descriptive statistics were employed to estimate the means and standard deviations of the study variables. After reliability testing, paired samples t-tests were conducted to observe the differences before and after using the instructional model. The relationships between the study variables were investigated using Pearson correlation analysis. The mediating effects of achievement emotions were tested using the PROCESS 4.1 plug-in. We used the Bootstrap technique to sample 5,000 times within the 95% confidence interval to test the significance of the direct, indirect, and total effects of the model. Linear regression analysis was used to analyze the causal relationships between variables. *p* < 0.05 was considered statistically significant.

### 2.6 Ethical considerations

Ethical application was approved by the Ethics Committee of the School of Nursing at the university (2024010801). The research followed all the ethical principles set out in the Declaration of Helsinki. The co-authors of this study explained the purpose and protocol of the study to the students prior to the distribution of the questionnaires. Informed consent from the students was obtained before they completed both questionnaires. Students were assured that their participation, or non-participation, would not affect their classroom performance.

## 3 Results

There were 87 nursing students in total who participated in the course. These students were all first-year master’s degree students in the Nursing School. Questionnaires completed by 64 participants (56 females and 8 males) were valid. All participants were aged between 21 and 25 years.

### 3.1 Preliminary analysis (Hypothesis 1)

Table 1 presents the descriptive statistical information, t-values, and Cronbach’s alphas for all variables. The results demonstrated high levels of control appraisals both before and after the instructional model, despite a decline at the second measurement (M1 = 3.94, SD1 = 0.53; M2 = 3.82, SD2 = 0.50).

Positive activating emotions (M1 = 3.89, SD1 = 0.37; M2 = 3.93, SD2 = 0.35), positive deactivating emotions (M1 = 3.53, SD1 = 0.43; M2 = 3.58, SD2 = 0.42), and negative activating emotions (M1 = 3.14, SD1 = 0.60; M2 = 3.12, SD2 = 0.64) were at higher levels both before and after the instructional model, while negative deactivating emotions (M1 = 2.61, SD1 = 0.57; M2 = 2.66, SD2 = 0.58) were at moderately low levels. Mean scores increased on all dimensions except for negative activating emotions, which decreased. In this teaching approach, students’ achievement emotions changed, supporting Hypothesis 1.

Furthermore, the mean scores for learning strategies were all at a high-level pre-instructional model and all improved post-instructional model (comprehension strategies: M1 = 2.79, SD1 = 0.44; M2 = 2.93, SD2 = 0.59; self-management strategies: M1 = 2.88, SD1 = 0.48; M2 = 2.93, SD2 = 0.51; effort level and persistence: M1 = 2.97, SD1 = 0.41; M2 = 3.02, SD2 = 0.49).

Lastly, students’ mean scores for attitudes (M1 = 4.02, SD1 = 0.32; M2 = 4.07, SD2 = 0.39), skills (M1 = 3.28, SD1 = 0.57; M2 = 3.42, SD2 = 0.57) and knowledge (M1 = 3.40, SD1 = 0.74; M2 = 3.80, SD2 = 0.56) improved from pre- to post-instructional model and were all on the high side. Also noteworthy was the significant growth in the skills (*p* < 0.05) and knowledge (*p* < 0.001) dimensions.

### 3.2 Correlation analysis between the variables (Hypothesis 2, 3)

Table 2 gives the correlations between the post-instructional model study variables. Control appraisals were significantly negatively correlated with negative activating (*r* = −0.31, *p* < 0.05), and deactivating emotions (*r* = −0.54, *p* < 0.001), while not significantly correlated with the other two emotional dimensions and learning strategies. Moreover, control appraisals were positively associated with attitudes (*r* = 0.50, *p* < 0.001) and were not significantly correlated with skills and knowledge. The correlations of control appraisals with achievement emotions, learning strategies, and evidence-based practice competencies did not fully hold, so Hypothesis 2 was partially supported. In the emotions and learning strategies dimension, only the correlation between positive deactivating emotions and effort level and persistence was significant (*r* = 0.25, *p* < 0.05). Positive activating emotions were positively correlated with attitudes (*r* = 0.33, *p* < 0.01), while positive deactivating emotions were not significantly related to attitudes. Negative activating (*r* = −0.27, *p* < 0.05) and deactivating emotions (*r* = −0.51, *p* < 0.001) were negatively correlated with attitudes. In addition, none of the dimensions of emotion were significantly correlated with skills and knowledge, so Hypothesis 3 partially supported. Regarding the relationship between learning strategies and evidence-based practice competencies, comprehension strategies (*r* = 0.27, *p* < 0.05) were positively correlated with knowledge. Besides, self-management strategies were positively related to attitudes (*r* = 0.27, *p* < 0.05) and knowledge (*r* = 0.35, *p* < 0.01), while no significant correlation was found with skills. Lastly, effort level and persistence were positively associated with attitudes (*r* = 0.37, *p* < 0.01), skills (*r* = 0.32, *p* < 0.01), and knowledge (*r* = 0.39, *p* < 0.01).

**Table 2:** Correlation between variables (*N* = 64).

Variable	CA	PA	PD	NA	ND	CS	SMS	ELP	EBPA	EBPS	EBPK
CA	–										
PA	−0.02	–									
PD	−0.19	0.80***	–								
NA	−0.31*	0.1	0.03	–							
ND	−0.54***	−0.34**	−0.23	0.70***	–						
CS	0.17	0.1	0.04	−0.05	−0.09	–					
SMS	0.24	0.12	−0.04	−0.02	−0.14	0.65***	–				
ELP	0.06	0.24	0.25*	−0.13	−0.22	0.63***	0.64***	–			
EBPA	0.50***	0.33**	0.22	−0.27*	−0.51***	0.22	0.27*	0.37**	–		
EBPS	0.11	0.19	0.19	−0.18	−0.24	0.21	0.18	0.32**	0.30*	–	
EBPK	0.09	0.2	0.21	−0.21	−0.23	0.27*	0.35**	0.39**	0.34**	0.60***	–
BM	−0.47***	−0.40**	−0.28*	0.60***	0.96***	−0.1	−0.16	−0.26*	−0.50***	−0.21	−0.18

*p < 0.05, **p < 0.01, ***p < 0.001.

### 3.3 The mediating effect of achievement emotions (Hypothesis 4)

**Figure 3:**
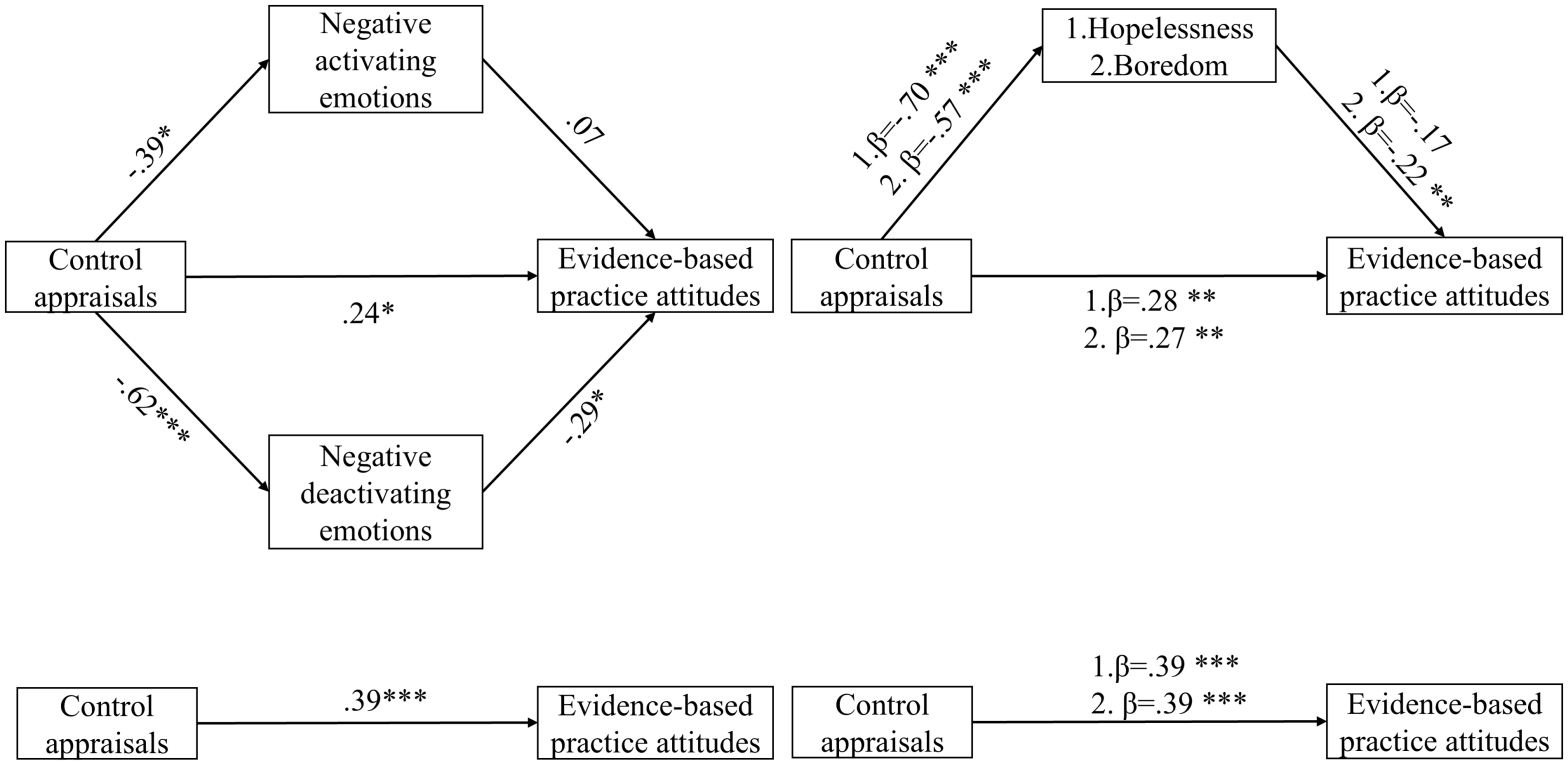
Mediating effects of achievement emotions between control appraisals and evidence-based practice attitudes. *p < 0.05, **p < 0.01, ***p < 0.001.

Since there was no correlation between control appraisals and learning strategies, we focused on the mediating effects among the other variables. Table 3 and Figure 3 demonstrate the fit indexes and coefficient significance for the mediating effects of achievement emotions between control appraisals and attitudes. Effect values for the mediation models of achievement emotions are shown in Table 4. We found that the path with negative activating emotions as a mediating variable did not hold (95%CI: −0.11, 0.06), whereas the path with negative deactivating emotions as the mediating variable did hold (95%CI: 0.00^a^, 0.36). Afterwards, we performed further tests and found that hopelessness as a mediating variable failed to hold (95%CI: −0.02, 0.27), while boredom did.

**Table 3:** Fit indexes and coefficient significance of the mediation models of achievement emotions (*N* = 64).

Variable		Fit index				Coefficient significance		
Outcome variable	Predictor variable	*R*	*R* ^2^	*F*	*P*	*β*	CI	*T*
CA → NA, ND → EBPA								
NA	CA	0.31	0.09	6.37	<0.05*	−0.39	−0.70, −0.08	−2.52*
ND	CA	0.54	0.30	26.03	<0.001***	−0.62	−0.87, −0.38	−5.10***
EBPA	CA	0.58	0.34	10.16	<0.001***	0.24	0.05, 0.44	2.46*
	NA					0.07	−0.11, 0.26	0.82
	ND					−0.29	−0.51, −0.06	−2.52*
CA → HE→EBPA								
HE	CA	0.58	0.34	31.39	<0.001***	−0.70	−0.95, −0.45	−5.60***
EBPA	CA	0.54	0.30	12.87	<0.001***	0.28	0.07, 0.48	2.68**
	HE					−0.17	−0.34, 0.00^a^	−1.95
CA → BM → EBPA								
BM	CA	0.47	0.22	17.77	<0.001***	−0.57	−0.83, −0.30	−4.22***
EBPA	CA	0.58	0.34	15.77	<0.001***	0.27	0.08, 0.45	2.92**
	BM					−0.22	−0.37, −0.07	−2.86**

*p < 0.05, **p < 0.01, ***p < 0.001. 0.00^a^ > 0.00.

**Table 4:** Effect values for the mediation models of achievement emotions (*N* = 64).

Model	Effect	Boot SE	CI	RE
CA → NA、ND → EBPA				
TE	0.39	0.09	0.22, 0.56	
DE	0.24	0.10	0.05, 0.44	61.54%
IE(NA)	−0.03	0.04	−0.11, 0.06	−7.69%
IE(ND)	0.18	0.09	0.00^a^, 0.36	46.15%
CA → HE→ EBPA				
TE	0.39	0.09	0.22, 0.56	
DE	0.28	0.10	0.07, 0.48	71.79%
IE	0.12	0.07	−0.02, 0.27	30.77%
CA → BM → EBPA				
TE	0.39	0.09	0.22, 0.56	
DE	0.27	0.09	0.08, 0.45	69.23%
IE	0.12	0.06	0.02, 0.27	30.77%

HE, hopelessness; TE, total effect; DE, direct effect; IE, indirect effect; RE, relative effect. 0.00^a^ > 0.00.

Boredom’s mean scores pre- and post-instructional model are displayed in Table 1. Boredom demonstrated a relatively low level (M1 = 2.57, SD1 = 0.59; M2 = 2.63, SD2 = 0.60) and increased after the instructional model. Table 2 provides the Pearson correlation between control appraisals, boredom, and attitudes. Control appraisals were negatively correlated with boredom (*r* = −0.47, *p* < 0.001), and positively correlated with attitudes (*r* = 0.50, *p* < 0.001), Boredom was negatively related to attitudes (*r* = −0.50, *p* < 0.001). As for the mediating effect, the indirect effect value for boredom was 0.12. The 95% confidence interval for the indirect effect of boredom was [0.02, 0.27], which did not contain 0, implying that the indirect effect held. Consequently, boredom played a mediating role in the model. The direct effect value for boredom was 0.27 with 95% confidence interval of [0.08, 0.45], which did not contain 0. Thus, boredom was a partial mediator between control appraisals and attitudes. Except for boredom, paths mediated by other individual emotions did not hold; therefore, Hypothesis 4 partially held true. Additionally, based on the calculation of the effect value, it can be concluded that the indirect effect of boredom accounted for 30.77%. The *F* value was 15.77 (*p* < 0.001), which showed that the model’s arithmetic results could truly and reliably reflect the effects of the independent and mediating variables on the dependent variable.

### 3.4 Relationship between control appraisals, learning strategies and evidence-based practice competencies (Hypothesis 5, 6)

As can be seen in Table 5, the test was conducted by setting attitudes as the independent variable and control appraisals as the dependent variable. The results indicated that attitudes had a significant positive influence on control appraisals (*β* = 0.50, *p* < 0.001) and that the independent variable explained 24% of the variance in the dependent variable. In contrast, learning strategies, knowledge and skills were not associated with control appraisals showed that Hypothesis 5 partially supported. The impact of learning strategies on evidence-based practice competencies was explored next. As for attitudes, only effort level and persistence (*β* = 0.33, *p* < 0.05) had an impact. In addition, effort level and persistence positively affected skills (*β* = 0.32, *p* < 0.01) but only explained 9% of the variance in the dependent variable. The rest could be explained by other variables outside the model. Lastly, none of the learning strategies had a significant effect on knowledge; thus, Hypothesis 6 was partially valid.

**Table 5:** The relationship between control appraisals, learning strategies, and evidence-based practice competencies (*N* = 64).

Dependent variable	Independent variable	*B*	*β*	*t*	*p*	Adjusted *R*^2^	F	*P*
CA	EBPA	0.64	0.50	4.58	<0.001***	0.24	20.97	<0.001***
EBPA	SMS	0.04	0.06	0.36	0.72	0.12	4.78	<0.05*
	ELP	0.26	0.33	2.13	<0.05*			
EBPS	ELP	0.38	0.32	2.70	<0.01**	0.09	7.27	<0.01**
EBPK	CS	−0.04	−0.05	−0.28	0.78	0.13	4.13	<0.01**
	SMS	0.21	0.20	1.17	0.25			
	ELP	0.33	0.29	1.78	0.08			

*p < 0.05, **p < 0.01, ***p < 0.001.

## 4 Discussion

To the best of our knowledge, this study was the first to explore the effectiveness of applying CBL within the CDIO framework. This instructional model provided a basis for how CBL can be applied to improve nursing students’ evidence-based practice competencies. Based on CVT, we also examined the relationship between control appraisals, achievement emotions, learning strategies, and evidence-based practice competencies in the context of an evidence-based nursing classroom that utilized this instructional model. The results of the study showed that boredom mediated the relationship between control appraisals and attitudes, and that attitudes positively predicted control appraisals, while effort level and persistence positively predicted attitudes and skills.

Although CBL has been applied to evidence-based practice education for a long time, research on how to standardize its application is lacking. At the same time, the implementation of evidence-based nursing is accompanied by inadequate skills among graduates and a disconnect between theoretical knowledge and practice, which can hinder the development of evidence-based careers. Regarding these issues, the instructional model used in this study provided a viable solution that could facilitate the improvement of knowledge and skills. This was consistent with previous findings (33–36) that CBL promoted higher levels of learning and assessment and enhanced students’ skills, and that the CDIO framework contributed to the development of high-level human resources and the enhancement of students’ self-directed learning. Moreover, the results also showed that nursing students held higher levels of positive achievement emotions. This indicated that the nursing students had a good experience with the instructional model used in this study. Therefore, diverse teaching strategies are highly appropriate for teaching evidence-based practice, which can benefit students in the learning process (37, 38). In conclusion, this model developed in this study, which combined real-life case scenarios with explicit implementation procedures, was eminently meaningful.

The CVT suggests that positive emotions such as pride depend on control appraisals, and that high control appraisals can motivate flexible learning strategies. However, in our study, control appraisals were only associated with negative emotions, but not with positive emotions and learning strategies. This was inconsistent with previous results. Future research should expand the sample for further measurements. In addition, control appraisals and emotions were only related to attitudes and not to knowledge and skills. We deduced that in the evidence-based nursing classroom, nursing students’ knowledge and skills are passively received, whereas their attitudes can be actively improved. Nursing students are more willing to take the initiative to learn when they believe they can accomplish the necessary learning tasks (39). Attitudes refer to the values ascribed by the learner to the importance and usefulness of evidence-based practice to inform clinical decision-making (9). Students in this study displayed high levels of attitudes both pre- and post-instructional model. High levels of attitudes reflected the enthusiasm and importance that students placed on evidence-based practice (40). Some scholars have also stated that attitudes are most closely related to evidence-based practice competencies compared to any other factor, and that their transformation can help promote real evidence-based change (41, 42). For these reasons, possessing high levels of attitudes is of paramount importance.

Our findings further indicated that boredom mediated the relationship between control appraisals and attitudes. This was consistent with previous findings that emotions mediated the relationship between appraisals and academic achievement (43, 44). Besides, as expected, control appraisals and attitudes were mutually causative. This demonstrated that the learning process for nursing students in the evidence-based nursing classroom was cyclical. Teachers can intervene at some point to improve nursing students’ evidence-based practice competencies. Boredom is a negative, deactivating emotion that is one of the most frequently reported emotions in the classroom and can be extremely destructive (45, 46). The detrimental effects of boredom on attitudes can impede the advancement of evidence-based nursing or result in unsound clinical judgments by nurses, ultimately compromising the delivery of safe and effective patient care. The CVT argues that boredom arises when perceived controllability is either too high or too low. In this study, we considered two factors that may contribute to students’ feelings of boredom. The first is that the difficulty of the selected task was inappropriate, making it either too challenging or too easy for students to complete the group assignments. The mismatch between individuals’ abilities and task requirements led to boredom among the students (47). The second factor is the teacher, who is at the core of the evidence-based nursing curriculum. Therefore, teachers need to do their best to enliven the classroom and increase student engagement in order to minimize students’ boredom (48). To address the boredom felt by students, we recommend incorporating elements of fun into course design as appropriate. Sabourin and Lester (49) suggested that game-based learning environments can keep students focused and increase their engagement and interest. Additionally, virtual reality (VR) technology can also be incorporated (50). The use of VR technology can help students shorten the gap between theory and practice, facilitate learning, and enable communication and interaction with virtual patients (51, 52).

Research on positive deactivating emotions is largely lacking. Our study confirmed that these emotions were positively correlated with effort level and persistence. Therefore, positive deactivating emotions can be beneficial for the development of students’ learning strategies in the evidence-based nursing classroom. The use of learning strategies is not only a sign of students’ active participation in the teaching-learning process, but it is also closely related to academic success and failure (53). Nursing students should be equipped with good learning strategies to meet various challenges. A survey of a course featuring evidence-based learning strategies (54) reported that students appreciated these strategies, and most felt that the course helped prepare them for clinical practice. We recognize that evidence-based learning strategies can foster critical thinking, improve students’ ability to ‘Seek and Use Evidence,’ and promote the development of evidence-based careers.

### 4.1 Limitations

This study was conducted within a single university in China, which not only resulted in a limited sample size but also constrained the generalizability of the findings to other cultural and educational contexts. Future research should aim to expand the sample size and adopt a multi-center design to further validate the universality of this study’s conclusions. Besides, limited course time may not allow instructors to answer students’ questions in a comprehensive and timely manner. To address this limitation, instructors could utilize platforms like WeChat in the future to facilitate real-time communication with students and assist in resolving their queries.

## 5 Conclusion

To effectively improve nursing students’ evidence-based practice competencies, this study designed the implementation steps of CBL based on the CDIO framework. The educational model was found to be showing promising momentum. If future educators apply this model in teaching and learning environments, it could produce even better evidence-based practitioners and contribute significantly to the development of evidence-based nursing. Moreover, although positive emotions prevailed in this study, negative emotions could not be overlooked. Consequently, we encouraged instructors to focus on students’ emotions in the learning environment to develop a better teaching atmosphere and enhance faculty-student interaction.

## Data Availability

The raw data supporting the conclusions of this article will be made available by the authors, without undue reservation.
